# Multimodal Analysis and Heterologous Expression Strategies Reveal the Crucial Role of the Lac2 Gene from *Lentinus sajor-caju* in Lignin Degradation

**DOI:** 10.3390/jof12090698

**Published:** 2026-09-17

**Authors:** Changlong Gou, Lilong Luo, Liang Zhang, Liming Chen, Yangci Liao, Yuqiong Wang

**Affiliations:** 1 College of Animal Science and Technology, Inner Mongolia Minzu University, 996 West Lamoulon Street, Tongliao 028000, China; 2 Institute of Pratacultural, Tibet Academy of Agricultural and Animal Husbandry Sciences, Lhasa 850000, China

**Keywords:** *Lentinus sajor-caju*, alkaline lignin, *Lac2*, overexpression

## Abstract

The complex chemical structure of plant lignin limits roughage utilization. Biological modification is commonly used to enhance lignin properties. *Lentinus sajor-caju* (*L. sajor-caju*) is a potent white-rot fungus for lignocellulose pretreatment in animal roughage. However, the key lignin-degrading enzyme genes remain unclear. This study evaluated the key enzyme genes involved in lignin degradation and their efficiency in decomposing alkaline lignin. Transcriptome and proteome comparative analyses of *L. sajor-caju* cultured in media with glucose and alkaline lignin as carbon sources revealed that the AA1 laccase aids lignin decomposition in *L. sajor-caju*. Additionally, a laccase heterologously expressing strain in *Pichia pastoris X-33* was constructed. Compared with the wild-type *L. sajor-caju* strain W-2, *Lac2* laccase activity increased by 67.35%. Electron microscopy showed that *Lac2* degraded alkaline lignin, forming irregular pores and surface fusion. These findings indicate that *Lac2* laccase has potential application value. In the future, it may be applied in agriculture, the chemical industry, and the energy sector to efficiently convert lignin into feed additives or energy products.

## 1. Introduction

As a major agricultural country, China yields a massive amount of straw, especially in Northeast China, the main grain-production region [1]. Straw typically contains 16–25% lignin and less than 5.5% crude protein on a dry matter basis, which restricts ruminal dry matter digestibility and leads to negative nitrogen balance in ruminants when used as feed [2]. Lignin in plants is composed of three basic structural units: p-hydroxyphenylpropane (H), guaiacyl (G), and syringyl (S) [3]. Each structural unit contains different functional groups on its benzene ring, and irreversible linkage results in a highly complex structural lignin [4]. The main linkages in lignin include β-O-4, β-5, β-β, and 5-5 bonds. The β-O-4 linkage accounts for approximately 50% of the total linkages and is a key bond targeted in lignin deconstruction. The structural characteristics and degradability of lignin vary according to its plant source: softwood lignin is predominantly composed of G units, hardwood lignin consists of a mixture of guaiacyl G and S units, and herbaceous plants contain a mixture of G, S and H units, resulting in a more complex structure [5]. The distribution of lignin is heterogeneous across different cell-wall layers (the middle lamella, primary wall, and secondary wall), cell types (fiber cells and vessel elements), and morphological regions, such as the lignin-enriched cell corners. Lignification begins in the cell corners and middle lamella and subsequently progresses into the secondary wall. The condensed lignin in the middle lamella is more difficult to remove [6]. Among the three methods for lignin degradation, the physical method requires a large amount of energy consumption, while the chemical method generates toxic residues. Fungal pretreatment is an efficient, economical, and environmentally friendly approach with low energy input for straw feed development and can effectively degrade lignin [7].

Based on differences in the attack modes and decomposition sites on wood during the degradation of wood components, fungi can be divided into three major categories: soft-rot fungi, brown-rot fungi and white-rot fungi [8]. White rot fungi strongly degrade lignocellulose and are widely used in various biotechnological industries, especially for the biological pretreatment of roughage utilization. Research shows that white rot fungi secrete enzymes, including laccase and class II peroxidases, which efficiently degrade lignin [9]. Based on their structure and function, Class-II peroxidases are classified into manganese peroxidase (MnPs), lignin peroxidase (LiPs), versatile peroxidase (VPs), and generic peroxidase (GPs) [10]. Laccase uses oxygen as an electron acceptor and typically oxidizes phenolic lignin structures directly, whereas class II peroxidases are heme-containing enzymes that rely on hydrogen peroxide to generate high oxidation potentials and can further attack nonphenolic lignin structures through radical-mediated reactions [11,12]. The complementary activities of laccase and hydrogen peroxide-dependent peroxidases enhance the efficiency of white-rot fungi in depolymerizing complex lignin polymers [13]. In recent years, multi-omics have been used to analyze *Pleurotus eryngii*-secreted laccases and constitutive/inducible aryl-alcohol oxidases (AAOs) on wheat straw at early fungal growth [14]. *Phlebia radiata* produces several abundant carbohydrate-active enzymes (CAZymes), including oxidoreductases, cellobiohydrolases, and multiple AA9 lytic polysaccharide monooxygenases on wood for 6 weeks [15]. *Pycnoporus coccineus* has been studied for enzymes involved in lignocellulose decomposition on pine and aspen [16]. This shows that oxidative enzymes play a key role in lignocellulose breakdown by white rot fungi. Laccases are Cu^2+^-containing phenol oxidases (multicopper oxidase family protein) and are especially widespread in white rot fungi [17]. They can oxidize a broad range of structurally diverse substrates—including monophenols, polyphenols, aminophenols, methoxyphenols, aromatic amines—and use molecular oxygen to form free radicals, which eventually reduce to H_2_O [18,19].

*Lentinus sajor-caju* is a commercially important edible mushroom in China. Previous research in our lab showed that *L. sajor-caju* efficiently degrades lignin [20]. *L. sajor-caju* is capable of degrading lignin via an enzymatic system comprising laccase and carboxymethylcellulase [21]. Studies have focused on the degradation rate of lignin, enzyme activity, and antioxidant activity. However, the key genes for *L. sajor-caju*’s lignin degradation remain unclear. *L. sajor-caju* can degrade lignin, making it a promising, safe biological feed for animal husbandry production. Therefore, this study aims to identify the key gene *Lac2* for lignin degradation through transcriptome and proteome analyses, and express it in the *Pichia pastoris* X-33 strain. The ability of *Lac2* to efficiently degrade alkaline lignin was verified through physical characterization and biochemical methods. The findings of this study could provide a theoretical foundation for the efficient utilization of lignin.

## 2. Materials and Methods

### 2.1. Microorganisms and Culture Conditions

The *Lentinus sajor-caju* strain W-2 was provided by the College of Animal Science and Technology of Inner Mongolia Minzu University. It was grown on yeast extract–glucose agar (YGA) slants and maintained at 4 °C. Alkaline lignin from Norway spruce (*Picea abies*) wood (CAS No. 8068-05-1; Catalog no. 370959, Sigma-Aldrich, St. Louis, MO, USA) was used. *Pichia pastoris* X-33 and the expression vector pPICZαA were procured from General Biol (Anhui, China). The fungi and culture medium were filtered through 40 µm cell strainers to collect fungal pellets. This fungus was inoculated into 100 mL of basal medium containing (*w*/*v*) 2% glucose, 2% malt extract, 0.1% peptone, 0.04% (NH_4_)_2_SO_4_, and 0.01% KH_2_PO_4_, and incubated at 25 °C for 7 days with shaking at 150 rpm. The collected pellets were washed thrice with sterile distilled water to remove residual media, then incubated at 25 °C for 7 days with shaking at 150 rpm. The fungal strain W-2 was inoculated into glucose–ammonium medium as a control. The glucose–ammonium medium contained (*w*/*v*) 1% glucose, 0.2% ammonium tartrate, 0.1% KH_2_PO_4_, 0.05% MgSO_4_,·7H_2_O, 0.05% KCl, 0.1% yeast extract and 1 mL/L trace elements solution, comprising 100 mg Na_2_B_4_O_7_·10H_2_O, 10 mg CuSO_4_·5H_2_O, 50 mg FeSO_4_·7H_2_O, 10 mg MnSO_4_·4H_2_O, 10 mg (NH_4_)_6_Mo_7_O_2_·4H_2_O, and 70 mg ZnSO_4_·7H_2_O. In the alkaline lignin–ammonium medium, glucose was replaced with an equal weight of alkaline lignin based on the glucose–ammonium medium. Alkaline lignin was sealed in sterile 300-mesh nylon bags and submerged in this liquid medium; the mesh permitted free diffusion of nutrients and extracellular enzymes but retained insoluble lignin particles within the bags. Three replicates of the glucose–ammonium and alkaline lignin–ammonium cultures were filtered. Total RNA was extracted from the mycelia, and the culture filtrates were employed for proteomic analysis.

### 2.2. Transcriptome Sequencing

The samples were sent to Personalbio Biotechnology Co., Ltd. (Shanghai, China), for total RNA extraction, RNA purification, reverse transcription, library preparation, and sequencing. Preparation and sequencing on the Illumina NovaSeq 6000 system (San Diego, CA, USA) using a 150 bp paired-end sequencing approach were performed for the cDNA libraries. Enrich the mRNA, then perform lysis with the lysis buffer. Synthesize the first-strand cDNA and synthesize the double-stranded cDNA using the SuperScript double-stranded cDNA synthesis kit (Invitrogen Life Technologies, Carlsbad, CA, USA). After quantification using the Illumina PCR Primer Cocktail in a 15-cycle PCR reaction, purification of the products was performed using the AMPure XP system, followed by quantification using the Agilent high-sensitivity DNA assay on an Agilent 2100 Bioanalyzer (Agilent Technologies, Palo Alto, CA, USA). The sequencing library was run on the Illumina NovaSeq 6000 platform.

The sequencing data were filtered using Cutadapt (v1.15) to obtain high-quality sequences (Clean Data) for further analysis. Bi-directional clustering analysis of differentially expressed genes (DEGs) was performed using the R language package Pheatmap (1.0.8). DEGs between groups were identified using thresholds of absolute fold change ≥ 2 and a false discovery rate (FDR) ≤ 0.01. The raw RNA-seq data of *Lentinus sajor-caju* (formerly *Pleurotus sajor-caju*) generated in this study have been deposited in NCBI Sequence Read Archive (SRA) under BioProject accession PRJNA1522565, with SRA run accessions SRR40490992, SRR40490991, SRR40490990, SRR40490989, SRR40490988 and SRR40490987.

### 2.3. Quantitative Real-Time PCR Analysis of Selected Ligninolytic Gene Expression

Quantitative real-time PCR (qRT-PCR) was performed to validate the relative transcriptional levels of selected ligninolytic genes identified as differentially expressed in the transcriptomic analysis. β-actin was used as the reference gene for *L. sajor-caju* W-2, and GAPDH was used for recombinant *Pichia pastoris*. The threshold cycle (2^−ΔΔCt^) method was employed to analyze relative gene expression; each transformant’s non-methanol-induced sample served as a calibrator, and three biological replicates were used, with calculations performed independently within each host.

### 2.4. Protein Extraction and Detection

The replicate samples were pooled, then freeze-dried. Sixty micrograms of protein were mixed with 5× SDT (4% SDS, 100 mM Tris-HCl, pH 7.6) buffer and boiled for 5 min. Proteins were separated by SDS–PAGE and stained with colloidal Coomassie blue. Protein bands were excised, and in-gel digestion was performed according to a standard trypsinolysis protocol [22]. Tryptic peptides were analyzed by timsTOF Pro mass spectrometry (MS) coupled to a NanoElute (Bruker, Billerica, MA, USA). Peptides were first loaded onto a C18-reversed phase analytical column (Thermo Scientific Easy Column, Madison, WI, USA, 25 cm long, 75 μm inner diameter, 1.9 μm resin) in 95% buffer A (0.1% formic acid in water). Separation was then achieved using a linear gradient of buffer B (99.9% acetonitrile and 0.1% formic acid) at a flow rate of 300 nl/min.

MS analysis was carried out in positive ion mode at an electrospray voltage of 1.5 kV, and fragment ions were detected with resolution across an *m*/*z* range of 100–1700. Operating in PASEF (parallel accumulation serial fragmentation) mode, the timsTOF Pro collected data with parameters including ion mobility coefficient (1/K0) set from 0.6 to 1.6 Vs·cm^2^, 1 MS and 10 MS/MS PASEF scans. Active exclusion was enabled with a 24 s release time.

MS data were analyzed using MaxQuant (v1.6.14; Max Planck Institute of Biochemistry, Martinsried, Germany) with standardized workflows. Precursor mass tolerance was set to 20 ppm, allowing a maximum of two missed cleavages. Carbamidomethylation of cysteines was set as a fixed modification, and methionine oxidation as a variable modification. The Q value (FDR) cutoff at the precursor and protein levels was ≤0.01. Protein sequences were searched using InterProScan (interproscan-5.25-64.0) to identify protein domain signatures from the Pfam database (v35.0).

Differentially expressed proteins (DEPs) were filtered with a fold change ≥ 2 or ≤0.5 and an unadjusted *p*-value ≤ 0.05. To identify key regulatory genes, we performed an interaction analysis between the top 10 CAZyme genes and DEPs. Initially, Pearson correlation coefficients were calculated from the expression abundance data of genes and proteins to evaluate their co-expression relationships, and significant gene-protein pairs were defined using thresholds of *p* < 0.05 and r > 0.8. The significantly correlated pairs were then uploaded to the omicshare online platform (www.omicshare.com/tools, accessed on 2 July 2026) to construct a gene-protein interaction network, in which nodes denote genes or proteins and edges represent significant correlations. Topological parameters, including degree centrality, were subsequently examined to identify genes with high connectivity, which were shortlisted as candidate hub genes that may play pivotal roles in the underlying biological processes. The mass spectrometry proteomics data have been deposited in the ProteomeXchange Consortium (https://proteomecentral.proteomexchange.org, accessed on 7 September 202) via the iProX partner repository with the dataset identifier PXD083781.

### 2.5. Plasmid Construction, Transformation, and Overexpression of Lac 2

The *Lac2* cDNA sequence and signal peptide length were retrieved from GenBank (GenBank: KAF4593541.1). The *Lac2* gene was amplified by PCR using the following primers: forward 5′-TATCTCTCGAGAAAAGA GAGGCTGAAGCTGCTATTGGTCCAGCTGGTAACATGTACATCGTCAAC-3′ and reverse 5′-AGGCTACAAACTCAATGATGATGATGATGATGGTCGACAGATGGAACGATACCACCTTTGT-3′. *Lac2* was digested with SaII and ligated into the pPICZα-A vector. The *Lac2*–pPICZα-A construct was transformed into competent *P. pastoris* X-33 by electroporation (4 kΩ, 50 µF, 400 V) using a Bio-Rad Gene Pulser, Hercules, CA, USA. Transformants were selected on YPD+Z plates (10 g/L yeast extract, 20 g/L peptone, 20 g/L glucose, 20 g/L agar, and 50 µg/mL Zeocin) and verified by colony PCR using primers 7059-F and 3′AOX. The selected clone was cultured in BMGY medium (10 g/L yeast extract, 20 g peptone, 13.2 g/L KH_2_PO_4_, 4 g/L (NH_4_)_2_HPO_4_, 1% glycerol, 0.5 g/L MgSO_4_, and 0.2mg/L biotin) and grown at 28 °C until OD600 reached 2–6. These cells were resuspended in 300 mL BMMY medium (20 g/L peptone, 10 g/L yeast extract, 13.4 g/L YNB, 0.2 mg/L biotin, 5 mL/L methanol and 0.1 M potassium phosphate buffer; pH 6.0) and incubated at 28 °C. The secondary culture was induced by adding 0.5% (*v*/*v*) methanol every 24 h. A 100 μL culture sample was centrifuged at 12,000 rpm for 5 min, and 80 μL of the supernatant was transferred to a 1.5 mL centrifuge tube. Twenty microliters of 5× loading buffer was added, and the samples were heated in a water bath for 10 min.

### 2.6. Characterization of Lignin and Enzyme Activity

Scanning electron microscopy (SEM) of treated and untreated lignin samples was performed at the Electron Microscope Facility. Small lignin pieces (2 × 2 mm) were fixed in 2.5% glutaraldehyde, dehydrated in an ascending acetone series, critical-point dried, and imaged with a scanning electron microscope (MIRA3, TESCAN, Brno, Czech Republic). Residual lignin content was determined gravimetrically by recovering, rinsing and drying the nylon bags to constant weight.

The fermentation broth was collected as the enzyme solution for analysis. One enzyme unit (U) is defined as the amount of enzyme that oxidizes 1 μmol of ABTS (2,2′-azino-bis (3-ethylbenzothiazoline-6-sulfonic acid)) per minute at pH 4.5 and 25 °C [23].

## 3. Results

### 3.1. Identification of Differentially Expressed Genes and Differentially Expressed Proteins Between the L. sajor-caju on Glucose–Ammonium and Alkaline Lignin Cultures

**Figure 1:**
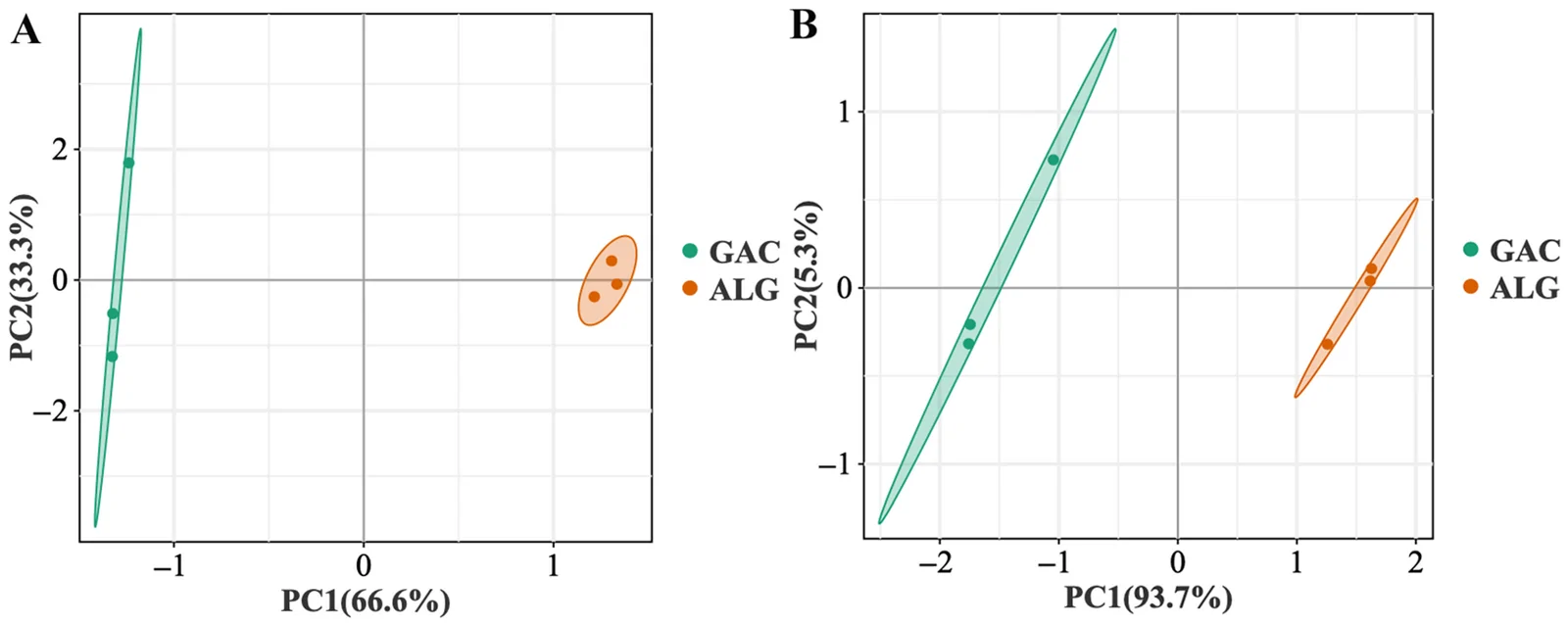
Principal component analysis of the log2-normalized RNA-seq read counts (**A**) and protein abundances (**B**) for six biological replicates of *L. sajor-caju* grown in alkaline lignin or glucoseammonium medium for 7 days. Replicates are shown as colored circles representing growth conditions: red for alkaline lignin and green for glucoseammonium medium.

**Figure 2:**
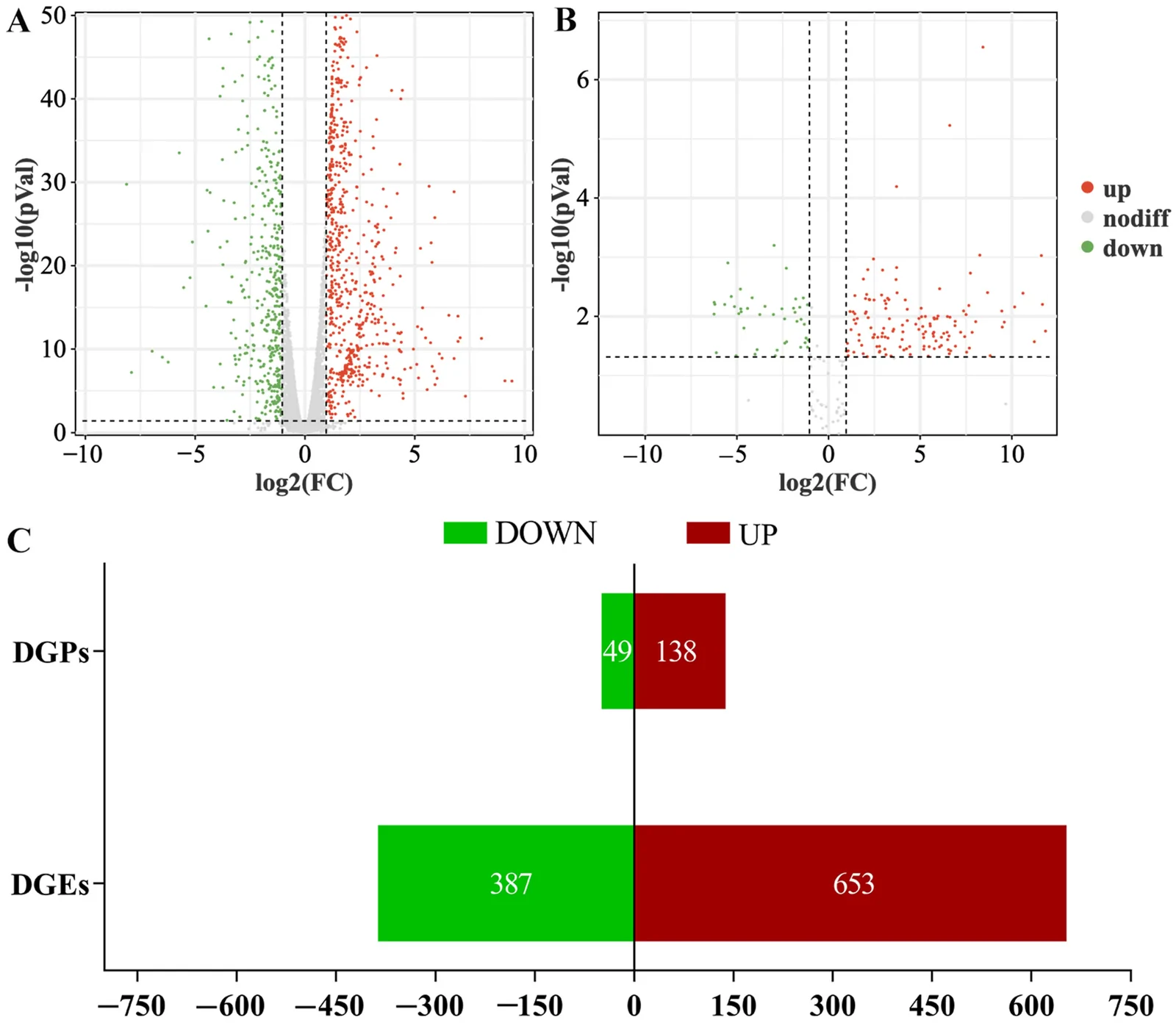
Analysis of differentially expressed genes and differentially expressed proteins. Volcano plots showing DEGs and DEPs in *L. sajor-caju* grown in alkaline lignin or glucoseammonium medium (**A**,**B**). Bar chart of the number of DEGs and DEPs between *L. sajor-caju* grown in alkaline lignin and glucose–ammonium medium (**C**).

Principal component analysis (PCA) showed that *L. sajor-caju* grown on glucose–ammonium clustered together, distinct from those of the alkaline lignin cultures (Figure 1). The PCA score plot shows a clear separation between GMC and ALC groups (Figure 1). Volcano plots from RNA-seq and label-free-based quantitative proteomics showed asymmetries of DEPs between down-regulated (green) and upregulated (red) proteins (Figure 2). These differences were attributed to *L. sajor-caju* exposure to alkaline lignin. These included 5687 DEGs in *L. sajor-caju* grown on lignin and glucose media. Among the DEGs, 653 were upregulated, and 387 were downregulated at day 7, based on a |log2 fold change (FC)| > 2 and *p*-value < 0.05. In addition, 221 differentially expressed proteins (DEPs) were identified (*p* < 0.05), with 183 proteins upregulated and 49 downregulated based on |log2 fold change (FC)| > 2 and *p*-value < 0.05, compared with those in the ALC group (Figure 2C).

### 3.2. Functional Analysis of Differentially Expressed Genes and Differentially Expressed Proteins

**Figure 3:**
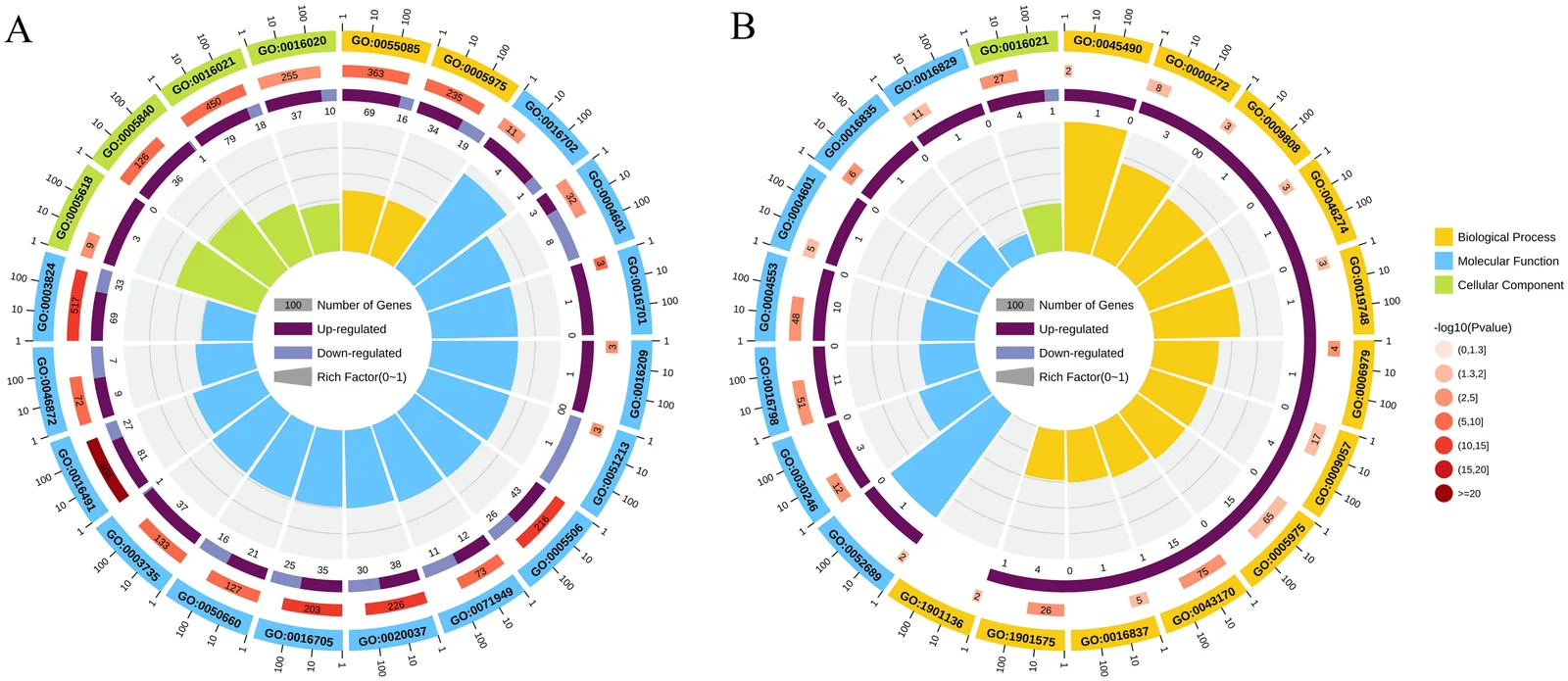
GO categories of differentially expressed genes and differentially expressed proteins. (**A**) GO enrichment analysis of DEGs (Top 20, *p* < 0.05); (**B**) GO enrichment analysis of DEPs (Top 20, *p* < 0.05).

The DEGs and DEPs were subjected to Gene Ontology (GO) functional cluster analysis to compare the ALC and GAM groups. Overall, 5687 genes and 221 proteins were classified into three GO categories: cellular component (CC), biological process (BP), and molecular function (MF). The top 20 GO items in each category, ranked by the smallest FDR, were statistically evaluated (Figure 3). GO enrichment analysis identified biological terms that were overrepresented among upregulated genes (|log2FC| ≥ 2 and FDR < 0.05) relative to their genomic proportion (Figure 3A). DEGs and DEPs were enriched in 466 and 417 GO items in the alkaline lignin and glucose–ammonium media, respectively. Among these, the numbers of items in the CC, BP, and MF categories were 36, 161, and 269 for DEGs, and 56, 239, and 122 for DEPs, respectively. GO enrichment analysis revealed that the most highly upregulated DEGs and DEPs were primarily associated with the oxidation–reduction process and carbohydrate metabolic process, respectively. Specifically, for the oxidation–reduction process, the enriched GO terms included “oxidoreductase activity” (GO:0016491) and “oxidoreductase activity, acting on paired donors, with incorporation or reduction of molecular oxygen” (GO:0016705). For carbohydrate metabolism, the enriched GO terms included “carbohydrate metabolic process” (GO:0005975), “catalytic activity” (GO:0003824), “heme binding” (GO:0020037), and “hydrolase activity, acting on glycosyl bonds” (GO:0004553). These terms are related to the metabolism of alkaline lignin and its associated degrading enzymes.

However, the downregulated DEGs and DEPs were mainly associated with catalytic activities related to peroxidase and lignin metabolism, including “peroxidase activity” (GO:0004601), “lignin catabolic process” (GO:0046274), “secondary metabolic process” (GO:0019748), and “lignin metabolic process” (GO:0009808).

### 3.3. CAZyme Genes and qRT-PCR

**Figure 4:**
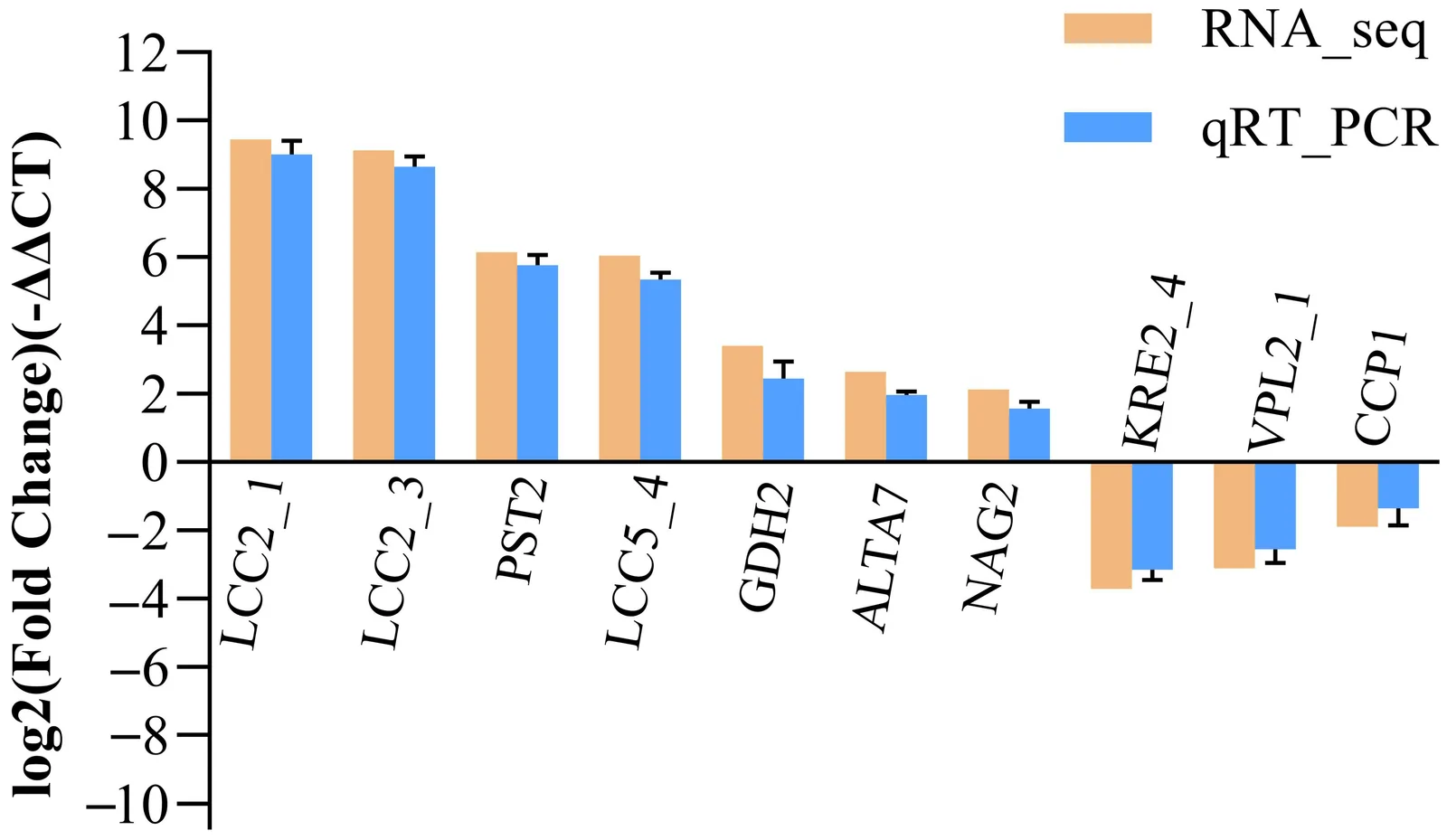
RNA-Seq and qRT-PCR data for the top 10 CAZyme genes.

Overall, 29 differentially expressed CAZyme genes were identified, including 4 glycosyl hydrolases (GHs), 2 carbohydrate esterases (CEs), 7 carbohydrate-binding modules (CBMs), 14 auxiliary activities (AAs), and 2 glycosyl transferases (GTs), all of which are involved in carbohydrate metabolism. Among the top 10 differentially expressed CAZyme genes, Laccase (AA1), glucose dehydrogenase (GDH2, AA3), protoplast secreted protein (PST2, AA6), minor allergen Alt a 7 (ALTA7, AA6), and beta-hexosaminidase (NAG2, CE9) were upregulated, whereas glycolipid 2-alpha-mannosyltransferase 2 (KRE2, GT15), versatile peroxidase (VPL2, AA2), and CCP1 (AA2) were downregulated. Based on the transcriptomic data (|log_2_ FC| ≥ 2), the top 10 differentially expressed CAZyme genes were validated via quantitative RT-PCR (qPCR) (Figure 4), which confirmed a high consistency with RNA-seq data (R^2^ = 0.992).

### 3.4. Construction of Gene-Weighted Co-Expression Network

**Figure 5:**
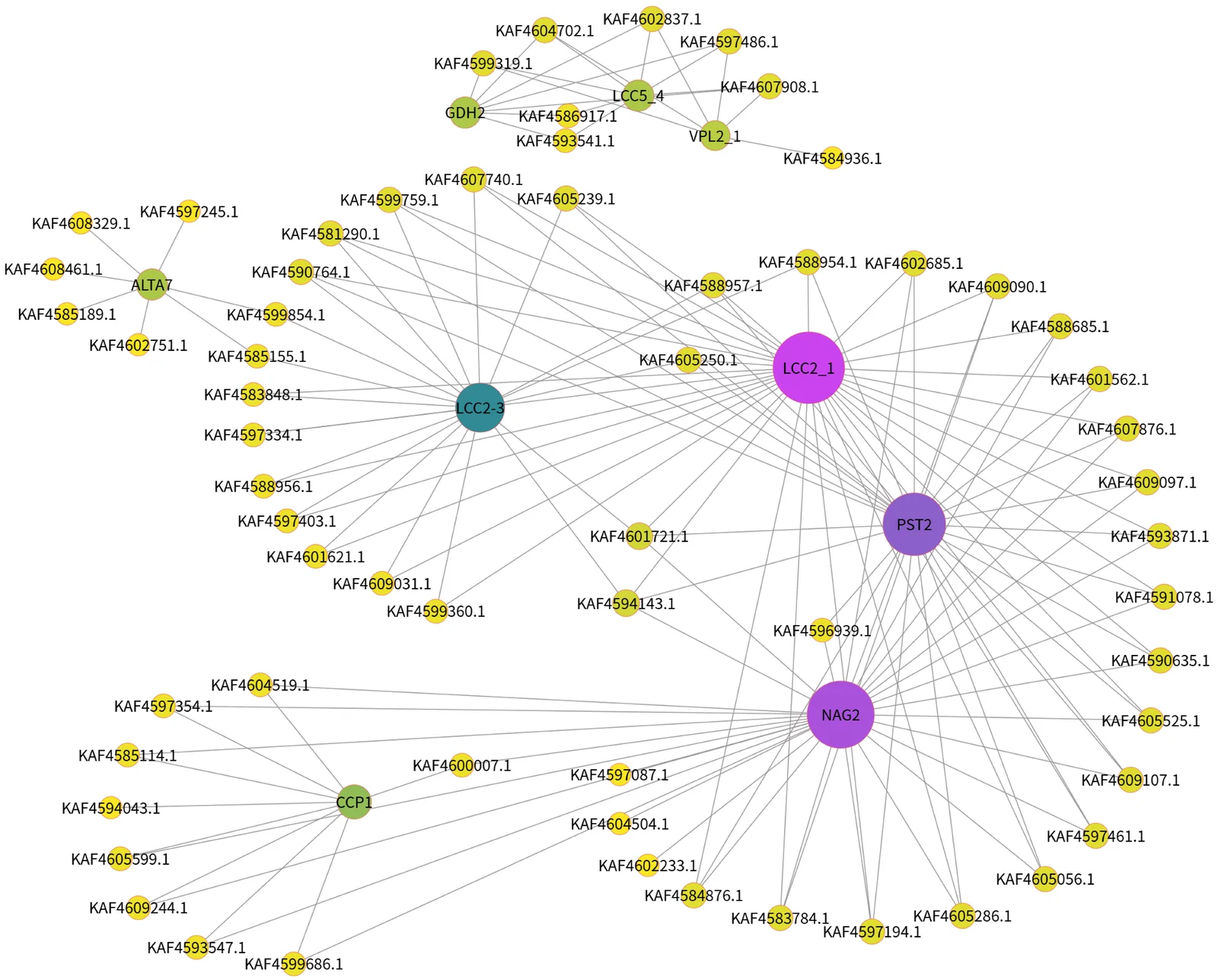
Interaction analysis of the top 10 CAZyme genes and DEPs.

The interactions between the top 10 CAZyme genes and DEPs (Figure 5). In the network, each node represents a gene, and the edges indicate regulatory relationships. Four key nodes were identified: *Lac2*_1, *Lac2*_3, PST2, and NAG2. Among these, *Lac2*_1 and *Lac2*_3 participate in the lignin decomposition process; PST2 was involved in the positive regulation of ferroptosis; and NAG2 was associated with glycan degradation. Overall, 40 proteins were associated with these four nodal genes, with *Lac2*_1 exhibiting the strongest associations.

**Figure 6:**
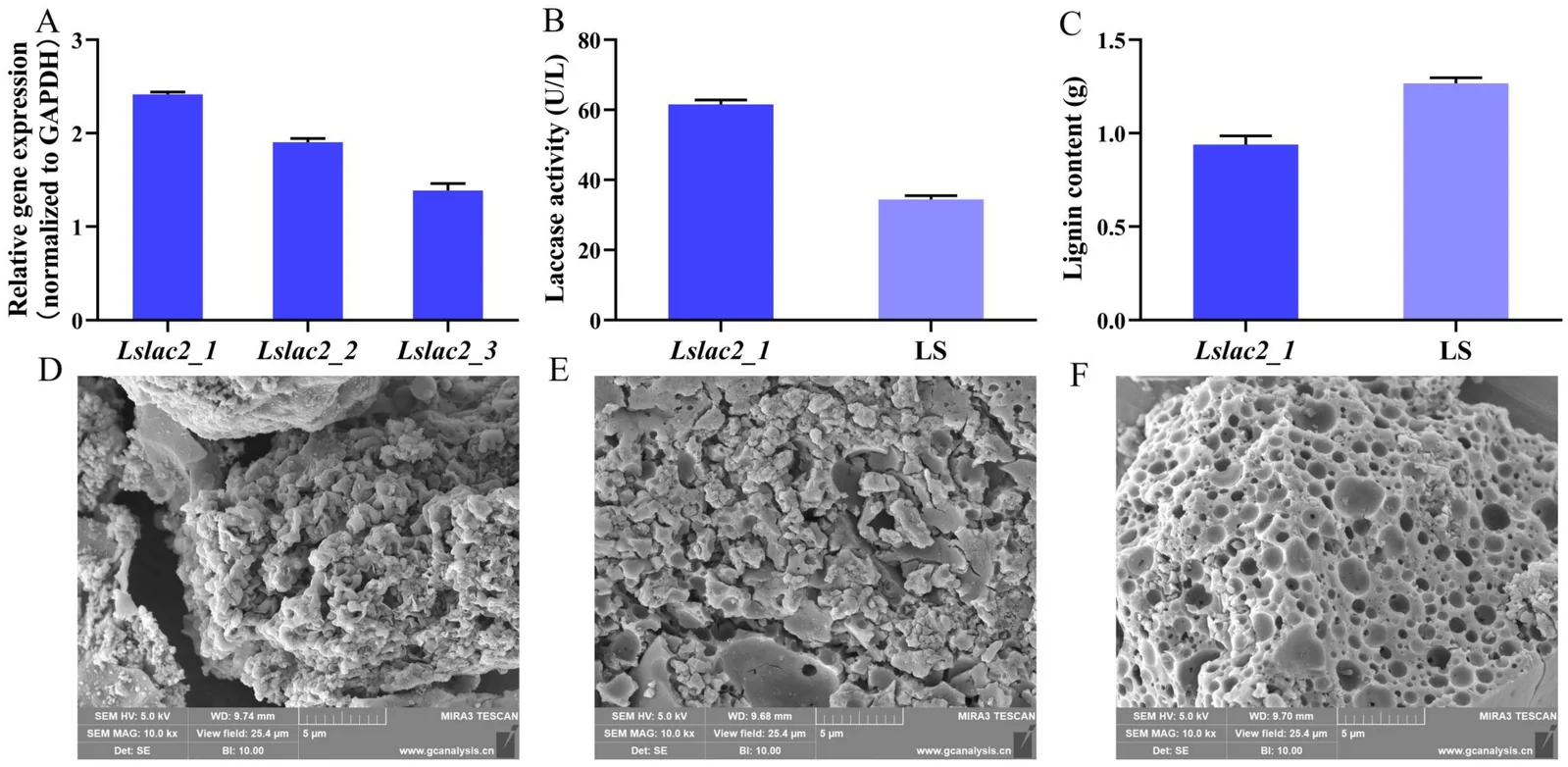
Comparison of traits between wild-type *L. sajor-caju* (LS) and recombinant *P. pastoris* strains overexpressing *Lslac2*. (**A**) Relative expression of *Lslac2*_1, *Lslac2*_2 and *Lslac2*_3 in the recombinant *P. pastoris* strains (normalized to GAPDH) after 7-day fermentation. Each transformant’s non-methanol-induced sample served as a calibrator, with three biological replicates. (**B**) Laccase activity of the wild-type LS and the *Lslac2*_1 recombinant *P. pastoris* strain after 7-day fermentation. (**C**) Residual lignin content in the medium after 7 days of fermentation by the wild-type LS and the *Lslac2*_1 recombinant *P. pastoris* strain. (**D**) Surface morphology of unfermented alkaline lignin. (**E**) Surface morphology of alkaline lignin after 7-day fermentation by the wild-type LS. (**F**) Surface morphology of alkaline lignin after 7-day fermentation by the *Lslac2*_1 recombinant *P. pastoris* strain. Note: the host-specific relative-expression values are not suitable for direct quantitative cross-species comparison.

The DNA of *L. sajor-caju* was amplified and sequenced, and the pPICzaA-*Polac2* expression construct was generated. The recombinant expression plasmid was integrated into the *Pichia pastoris* genome, resulting in the x33-po*lac 2* strain. Three stable genetic transformants, designated *Lslac2*_1–3, were obtained. Among them, the *Lslac2*_1 strain exhibited the highest expression level and was selected for subsequent studies (Figure 6A). After 7 days of fermentation, the laccase activity of the *Lslac2*_1 recombinant *P. pastoris* strain and wild-type *L. sajor-caju* strain were 61.55 and 34.45 U/L, respectively (Figure 6B). The laccase activity of *Lslac2*_1 recombinant *P. pastoris* was 1.79-fold higher than that of the wild-type *L. sajor-caju* strain, which may contribute to improved lignin-degrading performance. Compared with unfermented alkaline lignin, the wild-type *L. sajor-caju* strain and *Lslac2*_1 recombinant *P. pastoris* strain significantly altered the apparent structure of alkaline lignin after 7 days of fermentation (Figure 6D,E). The unfermented alkaline lignin showed distinct particles, defined surface topography, and interparticle linkages. After fermentation with the strains, the lignin exhibited a flattened morphology, characterized by irregularly sized circular cavities and concave surfaces. Fermentation by the wild-type strain disrupted the lignin structure, resulting in its disintegration into irregular particles with varying degrees of circular depression. Compared with type *L. sajor-caju*, the *Lslac2*_1 recombinant *P. pastoris* strain had larger cavities and enhanced lignin depolymerization, forming interconnected lignin networks.

## 4. Discussion

### 4.1. Transcriptome and Proteomics Analysis

Lignin is the most recalcitrant component of plant cell walls and poses a major barrier to biomass degradation. Through the secretion of diverse extracellular ligninolytic enzymes, white-rot fungi possess a unique capacity to degrade lignin [24]. Following lignin degradation, cellulose and hemicellulose become more accessible, thereby facilitating their efficient conversion into energy, livestock feed, and other value-added products. In recent years, transcriptomic and proteomic approaches have been increasingly applied to investigate fungal fermentation processes and lignin degradation. Lignin degradation by white-rot fungi mainly depends on an enzymatic oxidative system, in which laccase, manganese peroxidase, and lignin peroxidase play key roles [25]. Mining key lignin-degrading enzyme genes using omics technologies enables a deeper understanding of lignin degradation mechanisms and facilitates advances in enzyme engineering and industrial biotechnology. These advances support the industrial application of ligninolytic enzymes in food processing, medicine, bioenergy production, papermaking, and agricultural waste treatment. Recent proteomic and transcriptomic studies have investigated the molecular basis of lignin-degrading enzyme systems in *Pleurotus eryngii* and *Pleurotus ostreatus* [14,26]. Previous studies have demonstrated that *L. sajor-caju* exhibits a strong capacity to valorize lignocellulosic substrates, with applications in environmental management [27], feed fermentation [28,29,30], and biofuel production [31]. Despite these advances, the lignin-degrading mechanism of *L. sajor-caju* remains poorly characterized, particularly from a comprehensive multi-omics perspective. In the present study, integrated proteomic and transcriptomic analyses were employed to investigate gene and protein expression responses during lignin biodegradation by *L. sajor-caju*. The results identify 5687 DEGs and 221 DEPs, thereby significantly advancing the understanding of lignin deconstruction in *L. sajor-caju*. Specifically, 653 DEGs are upregulated and 387 are downregulated, while 183 DEPs are upregulated and 49 are downregulated between the ALC and GAM groups [|log2 fold change (FC)| > 2]. Moreover, gene expression at the transcriptional and protein levels is often asynchronous [32], which is consistent with that of Desgagné-Penix [33]. Transcriptomic and proteomic analyses consistently demonstrate an imbalance between transcriptional activity and protein abundance, indicating that mRNA levels do not always directly reflect protein expression. This discrepancy is likely driven by multiple post-transcriptional regulatory mechanisms, underscoring the critical role of post-transcriptional regulation in fungal lignin degradation. Proteomic analyses further reveal substantial variations in protein abundance in white-rot fungi grown on distinct lignocellulosic substrates. Because lignin depolymerization is inherently an extracellular process [34], related studies predominantly focus on the analysis of extracellular proteins. In the present study, 221 proteins were identified in the extracellular proteome. Specifically, 793 proteins were detected in *Phanerochaete chrysosporium* cultured on different poplar varieties [35], 72 proteins in *Postia placenta* grown on aspen [36], 897 proteins in *Pleurotus eryngii* cultured on wheat straw [14], and 434 proteins in *P. ostreatus* grown on poplar wood and wheat straw [26]. Consequently, elucidating the exact functions of extracellular proteins in white-rot fungi during lignocellulose decomposition remains an important objective for future research.

In this study, DEGs and DEPs were enriched in GO terms related to oxidoreductase activity (GO:001649, GO:0055085, GO:0016021, and GO:0003824; 392 DEGs) and carbohydrate metabolic process (GO:0005975, GO:0043170, GO:0016798, and GO:0004553; 51 DEPs). GO enrichment analysis indicates that differentially expressed genes in *L. sajor-caju* in response to alkaline lignin mainly mediate transmembrane carbohydrate transport and encode enzymes involved in lignin oxidation–reduction reactions. In contrast, the differentially expressed proteins predominantly function as enzymes involved in carbohydrate metabolism and the hydrolysis of glycosidic bonds. Collectively, these findings indicate that DEGs and DEPs enriched in these GO categories play a critical role in the lignin deconstruction capacity of *L. sajor-caju*.

### 4.2. Carbohydrate-Active Enzymes in L. sajor-caju

Carbohydrate-active enzyme (CAZymes) mainly include glycosyltransferases (GTs), glycoside hydrolases (GHs), auxiliary activity enzymes (AAs), carbohydrate esterases (CEs), and carbohydrate complex recognition (carbohydrate binding modules [CBM]). These enzymes participate in the biological transformation of carbohydrates and carbohydrate-derived compounds. The white-rot fungi secrete a diverse array of oxidative enzymes that enable the depolymerization of lignin fibers. Most of these lignin-degrading enzymes are classified within the carbohydrate-active enzyme (CAZymes) database [26], facilitating the rapid identification and functional annotation of carbohydrate-active enzymes. In this study, the secreted proteins of *L. sajor-caju* grown on glucose–ammonium and alkaline lignin–ammonium medium are compared, with particular emphasis on CAZymes profiles.

The composition and abundance of CAZymes secreted by white-rot fungi vary depending on the carbon source present in the culture media. When lignocellulosic substrates are used as the carbon source, oxidoreductases are highly abundant, whereas their abundance is markedly reduced in glucose-based media [26]. In this study, the extracellular CAZymes secreted by *L. sajor-caju* grown on alkaline lignin mainly comprise 14 AAs, 7 CBMs, 4 GHs, 2 CEs, and 2 GTs, with AAs accounting for 48.27%. Under solid-state culture conditions using wheat straw, although the number of extracellular protein AAs increases in *Pleurotus eryngii*, the content of GHs is the highest, reaching approximately 2.2 times that of AAs [14], which contrasts with the findings of the present study. The GHs belong to the glycoside hydrolases and mainly participate in cellulose degradation [37]. Under culture conditions containing lignocellulose substrates, these enzymes are typically produced in large quantities [38]. In this study, compared with the glucose medium, the expression of AAs in *L. sajor-caju* cultured in alkaline lignin medium is higher than that of other CAZyme classes; however, GHs do not show significant changes in the lignin medium. Overall, different lignocellulosic substrates exert a profound influence on enzyme production in white-rot fungi.

### 4.3. Laccases in the L. sajor-caju

Laccase is one of the earlier-appearing metalloproteins. Its lignin-degrading function arises from substrate specialization [39]. Laccase belongs to the AA1 family of CAZymes and catalyzes the oxidation of phenolic lignin and phydroxybenzoic acid, thereby generating phenoxyl radicals. These intermediate products serve as reaction mediators, facilitating lignin depolymerization [40]. While laccase is produced by diverse sources, ascomycete fungi are recognized as the main producers [41]. *Pycnoporus cinnabarinus* secretes laccase as the main extracellular protein in lignocellulose-based liquid culture and solid-state fermentation [40]. When cultured in a medium containing lignocellulose, *Pleurotus ostreatus* generates substantial quantities of four laccase enzymes: *Lac2*, *Lac6*, *Lac9*, and *Lac10*. Among these, *Lac10* is the predominant protein in poplar wood and wheat straw culture media [26]. Previous proteomic studies on white-rot fungi have identified laccases and peroxidases; however, laccase yield varies in response to environmental factors [42]. Laccase gene functions differ across organisms, making research on specific laccase genes of the target strain essential for their effective industrial application. In this study, within the AA class, six AA1, three AA2, one AA3, two AA6, and two AA9 enzymes were identified. AA1 accounted for 46.15%, mainly including *Lac2*_1, *Lac2*_3, *Lac5*_2, *Lac5*_4, *Lac5*_3, and *Lac5*_6. Compared with that of the glucose culture medium, the production of *L. sajor-caju* in the lignin-containing medium decreased for *Lac5*_2, *Lac5*_3, and *Lac5*_6, while the *Lac2*_1 production increased the most. Furthermore, RNA-seq and proteomic analyses identified key *Lac2* genes for functional verification, confirming that *Lac2* facilitates lignin degradation by *L. sajor-caju*. Following functional validation of *Lac2*, subsequent studies can investigate its synergistic interactions with other enzyme systems, aiming to construct a more efficient lignin-degradation platform and ultimately harness its potential for sustainable industrial applications.

Overall, overexpression of *Lac2* contributed to more efficient degradation of alkaline lignin by *L. sajor-caju*. Future research could consider optimizing the enzyme-producing conditions and the feasibility of scaling up production.

## 5. Conclusions

Comparative analysis of substances secreted by *L. sajor-caju* in alkaline lignin and glucose culture media revealed that CAZymes—including AAs, CBMs, GHs, CEs, and GTs—participate in lignin degradation under alkaline lignin conditions. *Lac2*_1 was identified as the key enzyme gene of *L. sajor-caju* responsible for alkaline lignin decomposition, and its efficient degradative activity was experimentally validated. These findings highlight the potential of *Lac2*_1 for the future development of novel laccases aimed at efficient lignin conversion.

## Data Availability

The original transcriptome data presented in the study are openly available in the NCBI SRA database under BioProject accession PRJNA1522565, with SRA run accessions SRR40490992, SRR40490991, SRR40490990, SRR40490989, SRR40490988 and SRR40490987, and the original proteomics data presented in this study are openly available in ProteomeXchange Consortium (https://proteomecentral.proteomexchange.org) with the dataset identifier PXD083781.

## Abbreviations

The following abbreviations are used in this manuscript:

GMC	Glucose–ammonium culture
ALC	Alkaline lignin culture
DEGs	Differentially expressed genes
DEPs	Differentially expressed proteins
GO	Gene ontology
CC	Cellular component
BP	Biological process
MF	Molecular function
FDR	False discovery rate
qRT-PCR	Quantitative real-time polymerase chain reaction
SDS-PAGE	Sodium dodecyl sulfate–polyacrylamide gel electrophoresis
CAZymes	Carbohydrate-active enzymes
GHs	Glycoside hydrolases
CEs	Carbohydrate esterases
CBMs	Carbohydrate-binding modules
AAs	Auxiliary activities
GTs	Glycosyl transferases
SEM	Scanning electron microscopy
MnPs	Manganese peroxidases
LiPs	Lignin peroxidases
VPs	Versatile peroxidases
